# Functional recovery after lumbar spinal stenosis surgery: Postural balance and residual urine as objective markers^☆^

**DOI:** 10.1016/j.bas.2025.104265

**Published:** 2025-04-28

**Authors:** Andreas K. Andresen, Leah Y. Carreon, Mikkel Østerheden Andersen

**Affiliations:** a Center for Spine Surgery and Research, Lillebaelt Hospital, Kolding, Denmark; b Institute of Regional Health Research, University of Southern Denmark, Winsløwparken 19, 3, DK-5000, Odense C, Denmark

**Keywords:** Degenerative, Lumbar spinal stenosis, Decompression, Balance, Residual urine, Outcome

## Abstract

**Introduction:**

Although the pathognomonic symptom of patients with lumbar spinal stenosis is impaired walking tolerance, they may also have subclinical impairment of postural balance and urinary retention due to chronic compression of the cauda equina.

**Research question:**

To investigate changes in postural balance and residual urine volume in patients with chronic lumbar spinal stenosis treated with decompression and fusion.

**Material and methods:**

The current study is a prospective longitudinal observational cohort study. Patients scheduled for decompression and fusion due to LSS with spondylolisthesis ≤ grade I were enrolled. Patient reported outcome measures, Tandem test for balance and post-voiding bladder ultrasound was performed at baseline and at 3, 12 and 24 months postoperatively.

**Results:**

Among 100 patients included, 90 % had complete follow up data available. The majority (77 %) were female with a mean age of 70.6 years. 89 % of patients reported symptoms for more than six months prior to surgery. All patient reported outcome measures were significantly improved after surgery. Tandem test scores improved from 20.0 at baseline to 26.6 at three months follow up (p < 0.001) which was sustained at two years. Clinically relevant residual urine volume (≥100 mL) seen in 12 patients pre-operatively was absent at two-year follow-up.

**Discussion and conclusion:**

Postural balance and post void residual urine, along with patient reported outcome measures, improved shortly after surgery, and the improvement was sustained two years after surgery. Physicians should consider using these two functional objective measures to evaluate disease severity and treatment effectiveness in patients with lumbar spinal stenosis.

## Highlights

• Decompression of the spinal canal improved postural balance and residual urine.
• Improvements were seen shortly after surgery, and sustained at two year follow up.
• Objective measures may guide clinicians to evaluate disease severity and outcome.

## 1 Introduction

Lumbar spinal stenosis (LSS) results in a reduced area of the spinal canal due to degenerative changes in the disc, the facet joints and buckling of the ligamentum flavum. Although the classical symptoms of LSS are sciatica and neurogenic claudication with decreased walking distance (Katz et al., 1994; Teske et al., 2015; Takahashi et al., 1995), a study by Iversen and Katz (2001) showed that 39 % of patients had a positive Romberg test indicating poor standing balance. Other studies comparing patients with spinal stenosis to asymptomatic controls found that the symptomatic patients showed significantly worse static balance (Truszczynska et al., 2014). This impairment in postural balance was present even in spinal stenosis patients who exhibited less severe symptoms (Huang et al., 2022).

LSS has also been suggested to cause urinary retention due to chronic compression of the cauda equina, with symptoms of detrusor underactivity, difficulty voiding completely and the need for double voiding (Panicker, 2020; Yamazaki et al., 2011). Earlier studies have investigated changes in urodynamics and found a significantly increased post-void residual urine in patients with spinal stenosis, compared with healthy controls (Cong et al., 2010). However, there have been no studies evaluating the impact of decompressive surgery on residual urine volume in patients with LSS.

The aim of the current study is to investigate changes in functional measures including tandem test for postural balance and residual urine volume in patients undergoing decompression and uninstrumented lumbar fusion for lumbar spinal stenosis.

## 2 Material and methods

The study was a single center, prospective longitudinal cohort study of patients undergoing lumbar decompression and fusion due to symptomatic spinal stenosis. A consecutive series of patients referred to Middelfart Hospital were screened for participation. Criterion for referral to the spine center was persistent symptoms after at least three months of non-operative treatment. All patients had Magnetic Resonance Imaging (MRI) performed to confirm the diagnosis of LSS. Patients were examined by a fellowship trained spine surgeon who had been in practice for at least five years. When the visit to the clinic was scheduled, patients underwent standing lumbar x-rays to rule out significant deformity of the spine. Patients with a greater than Meyerding Grade I (Koslosky and Gendelberg, 2020; Spondylolisthesis, 1932) spondylolisthesis or lumbar degenerative scoliosis more than 20° were excluded.

Inclusion criteria were patients aged 60 and above, no signs or history of neurological disease, significant spinal stenosis at one or two levels from L1 to S1 verified on MRI, neurologic claudication with a Konno score of >6 (Konno et al., 2007) and completion of a minimum of 3 month of conservative treatment without improvement of symptoms.

Approval for the study was obtained from the Regional Committees on Health Research Ethics for Southern Denmark (S-20120012). Data use approval was required from the Danish Data Protection Agency ref nr: 16/1586.

### 2.1 Treatment

Standard practice for elderly patients (age 60+) with Meyerding grade I spondylolisthesis was uninstrumented fusion, this was chosen due to a possible increased risk of screw loosening due to poor bone stock.

All patients underwent midline-sparing decompression with removal of the ligamentum flavum and resection of 1/3 of the hypertrophic facet joints. The posterolateral gutters were prepared by decorticating the lamina to the tip of the transverse process and the lateral facet. Local harvested allograft was mixed with allograft bone obtained from fresh frozen femoral heads and was distributed evenly among the gutters.

At 3 months follow up, the primary surgeon examined all patients and after this visit they started rehabilitation in the municipality. At 12 months post-operative, the patients underwent clinical reexamination, which included standing X-rays and a CT-scan.

### 2.2 Outcome measures

Demographic data regarding age, gender, height, weight, and comorbidities were collected from questionnaires and electronic patient records. Surgical data, length of stay and perioperative complications were collected by a research nurse at surgery and at discharge from the inpatient clinic. Patient reported outcomes on back- and leg pain were measured on a 0–100 visual analogue scale (VAS) (Williamson and Hoggart, 2005), where higher scores reflect more severe pain.

Secondary outcome measures were the Oswestry Disability Index (ODI), which measures spine related disability on a scale from 0 to 100, where higher scores reflect more severe disability (Lauridsen et al., 2006). Health related quality of life (HRQL) was measured by the EuroQol-5D-3L (EQ-5D), which is a preference-based measure with questions on mobility, self-care, activities, pain and anxiety/depression. EQ-5D is converted into an index score where 0 equals’ death and 1 equals perfect health (EuroQol, 1990; Sorensen et al., 2009).

### 2.3 Tandem test

**Fig. 1:**
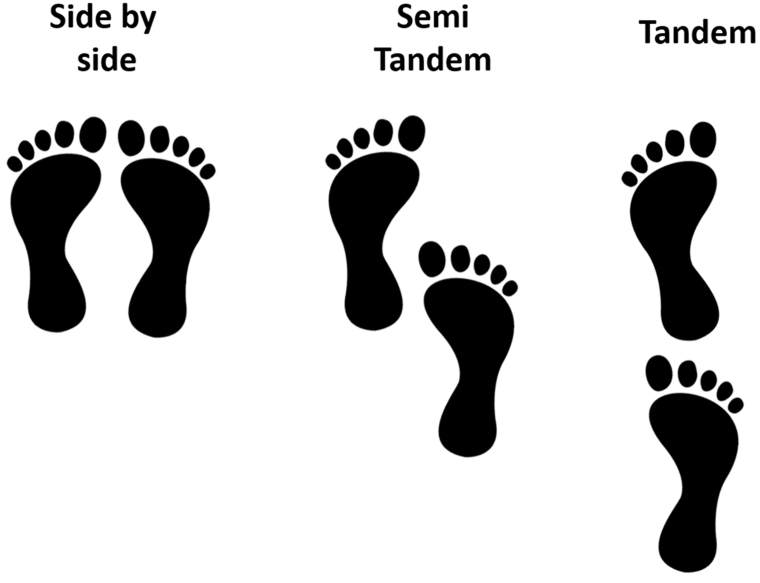
Tandem test standing positions.

The tandem test as described by Guralnic (Guralnik et al., 1995) was used to test postural balance. The test measures balance on a scale from 0 to 30, where higher scores reflects better postural balance. The test is performed in 3 steps, where patients can only progress to the next step if they score the maximum possible at the prior step. The test is performed with eyes open and arms hanging at their sides. Part 1 of the test is standing with feet parallel, one point is awarded per second until a maximum of 10 points. Part 2 of the test is standing in semi-tandem, again patients score points based on how many seconds they can stand in semi-tandem. Part 3 of the test is with both feet in tandem position (Fig. 1). After each step, patient is allowed to sit down to ensure that loss of balance is not due to fatigue. The test is ended if the patient moves a foot to retain balance.

Test scores from part 1, 2 and 3 are combined for a total score (0–30). The test was performed preoperatively and repeated at 3 months, 12 months and 24 months follow up. Test scores were summarized by the assisting physiotherapist.

#### 2.3.1 Bladder volume

Post-void bladder urine volume was measured in the outpatient clinic using the same ultrasound bladder scanner (BioCon-700 Mcube Technology co., Ltd.) and a standard protocol. Patients were instructed to attempt double voiding prior to the scan. The examination was performed with patients resting in supine position. The scan was performed 3 times, and mean value was noted. The test was performed at study inclusion and at 3, 12 and 24 months follow up. The test was conducted by a registered nurse, trained in ultrasonic bladder scanning protocols. Based on earlier studies, bladder volume of 100 ml was defined as the threshold for an abnormal Post-void Residual Urine Volume in older patients (Kelly, 2004; Sakakibara et al., 2005).

### 2.4 Statistics

All continuous variables were tested for normality using the Shapiro-Wilk test. Pre and post-variables were compared using paired *t*-test for normally distributed continuous variables, presented as means and 95 % confidence intervals, Wilcoxon rank was used for non-normally distributed continuous variables and Fisher's exact test for categorical data, presented as numbers and percentages. Statistical analyses were performed in STATA 18.0 (StataCorpLLC, College Station, TX) with a p-value of <0.05 considered statistically significant.

## 3 Results

**Fig. 2:**
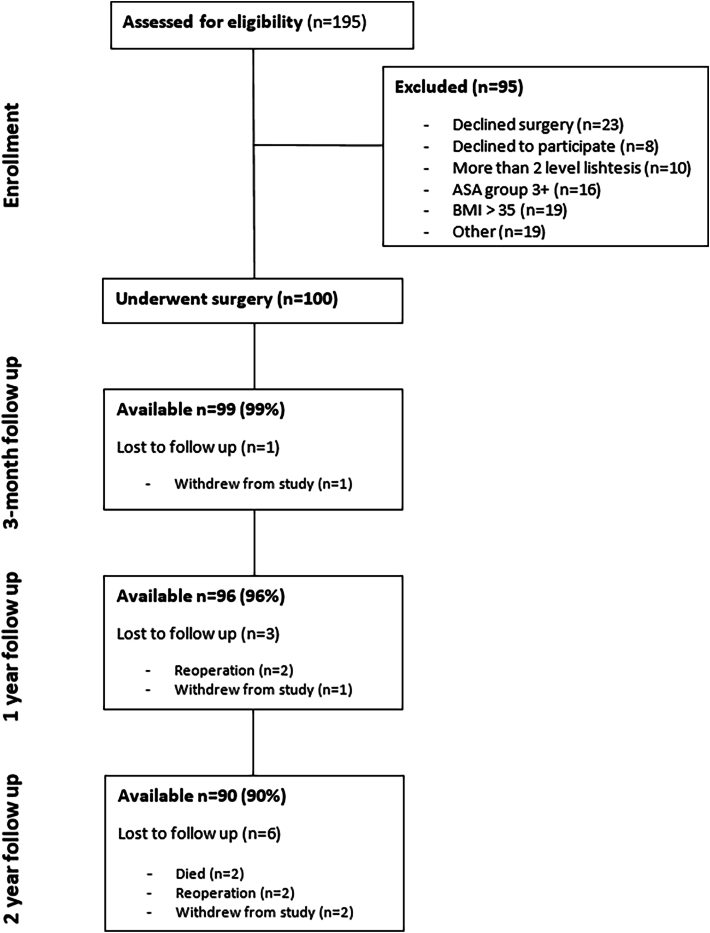
Flowchart of patient allocation and follow up.

100 patients were included in the study, mean age at inclusion was 70.6 years, 77 % were female, and 89 % of the patients had symptoms for more than 6 months prior to surgery (Table 1). At two years after surgery, 90 patients had complete follow up data available and were included in the analyses. (Fig. 2).

**Table 1:** Baseline characteristics of patients at inclusion. ∗BMI = Body Mass Index.

Patient characteristics	n = 90
Age, years, mean (SD)	70.6 (6.3)
Females, n (%)	69 (77 %)
BMI∗, kg/m^2^, mean (SD)	26.9 (3.9)
Hypertension, n (%)	47 (52 %)
Diabetes Mellitus, n (%)	10 (11 %)
Duration of symptoms, n (%)	
3 – <6 months	10 (11 %)
≥ 6–12 months	21 (23 %)
≥ 12 months	59 (66 %)

Significant improvements were observed from the preoperative period to 2 year follow up on both back and leg pain. Back pain improved by 37.6 points (95 % CI, [31.8; 43.4], p < 0.001) and leg pain by 44.3 points (95 % CI, [38.2; 50.2], p < 0.001). The mean improvement in ODI was 21.2 points (95 % CI, [17.9; 24.5], p < 0.001). Quality of life as measured by the EQ-5D showed a similar improvement of 0.28 points (95 % CI, [0.23; 0.32], p < 0.001) (Table 2).

**Table 2:** Summary of functional outcome measures. ∗ANOVA repeated measures. #Chi2 test.

	Preoperative (n = 90)	3 months Post-operative (n = 90)	12 months Post-operative (n = 90)	24-months Post-operative (n = 90)	p-value
Oswestry Disability Index, mean (SD)	38.8 (13.9)	19.0 (15.0)	16.6 (14.9)	17.2 (16.5)	<0.001∗
EuroQol-5D, mean (SD)	0.54 (0.21)	0.78 (0.15)	0.82 (0.16)	0.83 (0.18)	<0.001∗
Visual Analogue Scale – Leg pain, mean (SD)	66.9 (18.3)	18.0 (21.8)	21.9 (26.5)	22.4 (26.8)	<0.001∗
Visual Analogue Scale – Back pain, mean (SD)	57.0 (24.5)	19.2 (17.6)	20.4 (23.1)	19.4 (24.0)	<0.001∗
Tandem test score, mean (SD)	20.0 (9.1)	26.6 (5.8)	27.0 (5.0)	27.0 (4.9)	<0.001∗
Post-Void Bladder Volume, ml, mean (SD)	39.9 (62.4)	20.3 (34.8)	16.2 (42.2)	9.9 (19.4)	<0.001∗
Proportion of subjects with Residual Bladder Volume >100 ml, N (%)	12 (13.3)	4 (4.4)	5 (5.6)	0 (−)	0.002#

A significant improvement in the Tandem test was found from pre-to postoperative at all follow up points. Preoperatively patients had a mean score of 20.0 points (95 % CI, [18.1; 21.9]), this improved by 6.6 points (95 % CI, [4.8; 8.6], p < 0.001) at three months post-operative. This improvement was sustained at 12- to 24 month follow up. Before surgery, 76 patients (84 %) reported a self-perceived wide-based gait, which was decreased to 14 patients (16 %) at two year follow up (Table 2).

**Fig. 3:**
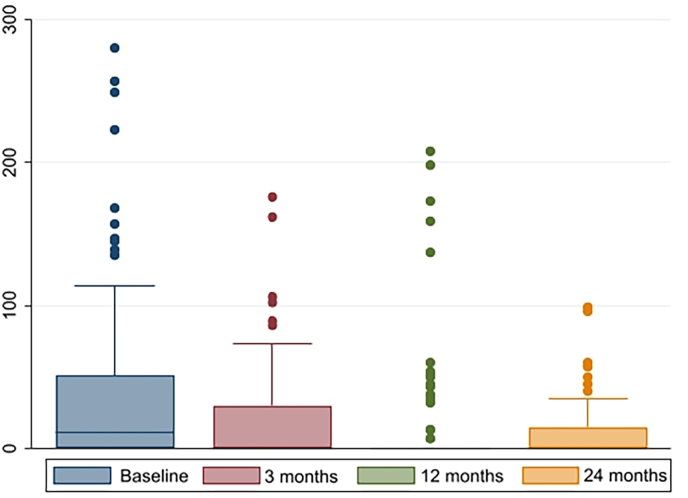
Post-void residual urine (mL).

Post-void residual bladder urine volume was 39.9 ml (95 % CI, [26.8; 53.1]) before surgery, with a mean improvement from baseline to 2-year follow up of 29.9 ml (95 % CI, [16.3; 43.9], p < 0.001). Twelve of the patients (13.3 %) had more than 100 ml post-void residual urine before surgery, this improved to four patients at three months follow up, five patients at 12 months and at two-year follow up, no patients had significant residual urine (Fig. 3). 17 (19 %) patients reported symptoms of lower urinary tract symptoms (LUTS) before surgery, the mean PVR was 67 ml (95 % CI, [26.4; 108.0]) in the LUTS group, versus 30 ml (95 % CI, [19.6; 40.9]) in the no-LUTS group p = 0.01.

## 4 Discussion

In this prospective cohort study of 100 patients with lumbar spinal stenosis, we found that patient reported outcome measures, objective measures for postural balance and post-void bladder volume were significantly improved after spinal decompression surgery. While the natural decline in walking capacity with aging is multifactorial and can be caused by decreased muscle strength, impaired proprioception, vision (Lord and Ward, 1994) and pain, problems with balance have been considered a primary factor in limiting the walking capacity in patients with spinal stenosis (Iversen and Katz, 2001).

We found scores for the Tandem Test to improve significantly from pre-operative to 24-months post-operative with the greatest improvement seen within three months after surgery. Thus, this improvement in postural balance is most likely attributed to the surgical decompression. Although specifically recognized as a tool in the context of spinal stenosis, the current study showed that it can be a useful adjunct to evaluate impairment in postural balance in patients with LSS and can be used as an objective measure of treatment effectiveness, offering valuable insights into a patient's functional status and aid in monitoring treatment outcomes.

This study also demonstrated significant reduction in both the mean post-void residual urine volume and the number of patients with pathological urine retention after decompressive surgery. In a study by Kimura et al. authors found that the prevalence of lower urinary tract symptoms was high in patients with spinal stenosis, with moderate symptoms present in 64 % of patients. They also found an increased number of patients with normal voiding patterns after decompression of the spinal canal at short term follow up (Kimura et al., 2022). In our current study we found a similar improvement in voiding patterns and fewer patients with significant residual urine volume after decompression of the spinal canal. In addition, the current study showed that his short term improvement persists 12–24 months after surgery.

Similar to numerous prior studies, we also found a significant improvement in all PROs in the current patient cohort after decompression surgery on all PROs from baseline to 3 month follow-up, this improvement was sustained at 2 year follow up.

The study does not include radiologic confirmation of adequate spinal decompression. However, given the lack of established correlation in the literature between spinal canal area and symptoms in patients with spinal stenosis before and after decompression surgery along with the significant improvement in self-reported pain scores from baseline to the 3-month follow-up—an improvement that was sustained at the 2-year follow-up—we believe the decompression was sufficient (Aaen et al., 2022a, 2022b; Hermansen et al., 2023).

Although the threshold value of 100 ml residual urine volume is seen as clinically relevant based on prior literature, the choice of another cutoff level may alter the results. In our cohort of 70 year olds, some patients may have prostate hypertrophy, cystocele or other urinary genital disorders that may result in increased residual urine. However our data suggest that chronic compression of the spinal cord may be part of the cause of urinary retention, and decompression seems to relieve the problem. As at the final follow-up no patient had a residual urine volume of more than 100 mL. The study was not randomized, and all patients were treated at a tertiary spine center where patients from the whole region are referred to treatment, and patients with lesser symptoms were screened and treated in primary and/or secondary care clinics.

## 5 Conclusion

In conclusion, we found that both measured static balance and urinary function improved shortly after decompression for spinal stenosis, this improvement was sustained two years after surgery. While these findings need confirmation in larger scale trials, residual urinevolume and balance may be considered an adjunct to PROMs when evaluating disease severity and treatment effectiveness in patients with lumbar spinal stenosis.

## Funding

No funding was acquired for the current study.

The Manuscript submitted does not contain information about medical device(s)/drug(s).

## Declaration of competing interest

The authors declare that they have no known competing financial interests or personal relationships that could have appeared to influence the work reported in this paper.
